# Biofunctionalization of mineralized collagen with platelet-rich plasma enhances osteogenesis in critical-sized bone defects

**DOI:** 10.3389/fbioe.2026.1877289

**Published:** 2026-07-17

**Authors:** Chong Gao, Yichen Wang, Minghui Qu, Cuihan Liu, Shisu Tao, Chenxiao Song, Jianwen Hou, Guangyun Hu, Feng Yang

**Affiliations:** 1 Department of Orthopedics, The Second People’s Hospital of Lianyungang Affiliated to Kangda College of Nanjing Medical University, Lianyungang, Jiangsu, China; 2 Department of Orthopedics, The Second Affiliated Hospital of Soochow University, Suzhou, Jiangsu, China; 3 Clinical Research Center of Neurological Disease, The Second Affiliated Hospital of Soochow University, Institution of Neuroscience, Soochow University, Suzhou, China; 4 Department of Orthopedics, Nantong Rici Hospital Affiliated to Yangzhou University, Nantong, Jiangsu, China

**Keywords:** bone marrow mesenchymal stem cells, bone regeneration, critical-sized bone defect, mineralized collagen, osteogenic differentiation, platelet-rich plasma, RUNX2

## Abstract

**Introduction:**

Bone defects caused by fractures, nonunion, tumors, or infections severely impair patient quality of life. Although autologous bone grafting remains the gold standard, its limited donor availability and additional surgical trauma have driven the development of biomimetic substitutes. Mineralized collagen (MC) replicates native bone extracellular matrix but lacks osteoinductive capacity. Platelet‐rich plasma (PRP) is a fibrin matrix enriched with growth factors (e.g., VEGF, PDGF, TGF‐β1, IGF‐1) that promote cell proliferation and osteoblastic differentiation, yet its gel‐like form suffers from poor structural integrity in bone defects.

**Methods:**

This study investigated whether the combination of PRP and MC produces enhanced effects on osteogenic differentiation of bone marrow mesenchymal stem cells (BMSCs) and healing of critical‐sized bone defects. PRP + MC composites were prepared by immersing MC scaffolds in PRP and evaluated using rat BMSCs *in vitro* (proliferation, ALP, Alizarin Red S, RUNX2 qRT‐PCR). *In vivo*, critical‐sized cylindrical femoral condyle defects (3 mm diameter, 5 mm depth) were created in male SD rats and filled with PRP + MC, MC, PRP, or normal saline. Bone regeneration was analyzed by Micro‐CT at 12 weeks, and histology was performed using H&E, Masson's trichrome, and OPN immunohistochemistry.

**Results:**

The PRP + MC composite significantly enhanced BMSC proliferation at day 7. ALP activity and mineralization were markedly increased, accompanied by significantly upregulated RUNX2 expression (∼5.6‐fold vs. control). Micro‐CT revealed near‐complete defect filling with high‐density trabecular architecture. Histologically, the composite group exhibited the highest new bone area (∼75%) and OPN expression, with mature lamellar bone and host integration, whereas controls remained dominated by fibrous scar tissue.

**Discussion:**

PRP + MC represents a biomimetic and cost‐effective strategy for enhancing bone repair. By combining structural scaffolding with biological stimulation, this composite activates RUNX2‐mediated signaling and achieves enhanced regeneration in critical‐sized defects, supporting its preclinical potential as an alternative to autologous bone grafting.

## 1 Introduction

Clinically, bone fractures, defects, and nonunions resulting from various pathogenic causes—such as trauma, tumors, and infections—are commonly encountered conditions that severely compromise patients’ quality of life while imposing substantial economic burdens on families and society (Sabouri et al., 2025). Autologous bone grafting, considered the “gold standard” for bone repair, presents several drawbacks, including the creation of additional surgical sites, prolonged operative time, increased blood loss, inability to provide specialized structural configurations, limited graft availability that fails to meet complex clinical requirements, restricted cortical bone supply, and potential complications such as donor site fractures when excessive bone harvesting is performed (Vermeu et al., 2022). Allogeneic bone grafts carry risks of disease transmission, immune rejection, inferior mechanical strength, and poor osteogenic capacity (Hou et al., 2023; Nguyen et al., 2022). Consequently, various metal, ceramic, polymeric materials, and their composites have been developed as bone substitutes (Wiącek et al., 2026; Fernandez de Grado et al., 2018). Research on bone substitute materials remains a focal point in tissue engineering and medical science.

Mineralized collagen (MC) has emerged as a biomimetic composite material that replicates the chemical composition and microstructure of natural bone matrix (Robin et al., 2024). MC consists of type I collagen and hydroxyapatite (HA), wherein the HA exists as weakly crystalline nanoparticles periodically arranged between collagen molecules (Dong et al., 2024). This biomimetic material shares identical chemical composition and microstructural characteristics with native human bone matrix, thereby providing a favorable microenvironment for osteocyte activity and facilitating guided bone tissue regeneration (Paulo et al., 2017). Animal experimental studies have demonstrated that MC effectively induces bone tissue regeneration and adequately repairs large segmental bone defects. Specifically, radiographic examination of rabbit radial defects (15 mm) at 12 weeks post-implantation revealed successful bone formation (Kim et al., 2017). Depending on the implantation site, the material degrades within 3–6 months *in vivo*, a timeframe that corresponds well with new bone formation and healing completion (Wüster et al., 2024). Clinical outcomes have also confirmed MC as an effective alternative to autologous bone grafts (Li et al., 2020). However, MC functions merely as a bone defect filler, lacking osteogenic cells and osteoinductive activity, which results in relatively slow bone formation rates (Zanca et al., 2022; Luo et al., 2023; Gao et al., 2020).

The incorporation of growth factors into bone substitutes has predominantly focused on single factors, such as bone morphogenetic protein (BMP) (Uijlenbroek et al., 2022). Nevertheless, following clinical introduction, BMP has failed to demonstrate significant efficacy in promoting bone repair in randomized controlled trials (Takase et al., 2024). Bone regeneration depends on the concentration and synergistic interactions of multiple growth factors within the microenvironment; thus, single growth factors often prove insufficient for effective bone repair (Song et al., 2023). Given that growth factors are primarily present in plasma and platelets, blood-derived extracts have garnered considerable attention (Dinulescu et al., 2024; Xie et al., 2022). Platelet-rich plasma (PRP), obtained through differential centrifugation, represents a fibrin matrix enriched with multiple high-concentration growth factors, including vascular endothelial growth factor (VEGF), fibroblast growth factor (FGF), platelet-derived growth factor (PDGF), epidermal growth factor (EGF), insulin-like growth factor-1 (IGF-1), transforming growth factor-β1 (TGF-β1), and bone morphogenetic proteins (BMPs) (Everts et al., 2020). These growth factors demonstrate favorable efficacy in bone tissue repair. PRP has achieved promising outcomes in oral and maxillofacial surgery applications (Masuki et al., 2016; Castro et al., 2024). However, some researchers remain skeptical regarding PRP efficacy. In its gel-like state, PRP exhibits soft consistency and poor structural integrity, rendering it ineffective for filling larger bone defects and prone to displacement or loss (Borsani et al., 2018; Van Lieshout and Den Hartog, 2021).

Therefore, we propose the combination of PRP and MC as a novel composite material and aim to validate its function in promoting the proliferation and differentiation of bone marrow mesenchymal stem cells (BMSCs) and facilitating bone defect repair through cellular and animal experiments.

## 2 Materials and methods

### 2.1 Materials and PRP preparation

#### 2.1.1 Preparation of mineralized collagen

Based on preliminary experimental results, mineralized collagen scaffold bone repair materials (Product batch number: G240520; Model: Re-7; Trade name: BonGold™ Mineralized Collagen Scaffold Bone Repair Material; Beijing Allgens Medical Science and Technology Co., Ltd., China) were selected for this study. The scaffold is composed of type I collagen and weakly crystalline nano-hydroxyapatite (nHA), with a porosity of approximately 70%–80% and pore sizes ranging from 200 to 400μm, as confirmed by SEM analysis. The mineralized collagen scaffold appeared as white porous cubes with a texture similar to plastic foam. The material was fabricated into cylindrical structures (3 mm in diameter, 5 mm in height) for *in vitro* cell culture or *in vivo* bone defect filling.

#### 2.1.2 Preparation of Platelet-Rich Plasma (PRP)

PRP was prepared from whole blood of SD rats using a two-step centrifugation method. Rats were anesthetized by intraperitoneal injection of 3% pentobarbital sodium (0.1 mL/100 g), and whole blood was collected via abdominal aortic puncture using anticoagulant tubes containing 3.8% sodium citrate (blood to anticoagulant volume ratio of 9:1). The whole blood was centrifuged at 200 *g* for 30 min at room temperature. After centrifugation, distinct layers were observed: the lower layer consisted of red blood cells, and the upper layer represented platelet-poor plasma. The upper plasma layer and the intermediate buffy coat were carefully aspirated and transferred to a new sterile centrifuge tube, avoiding aspiration of the red blood cell layer. The collected plasma was then centrifuged again at 2000 × g for 10 min. Most of the supernatant (platelet-poor plasma) was discarded, leaving approximately one-fifth of the volume at the bottom of the tube. The precipitate was gently resuspended to obtain PRP (Jiang et al., 2022).

Before use, an aliquot of each PRP batch was characterized by manual counting using a Neubauer hemocytometer. The acceptance criteria were defined as: platelet concentration ≥800 × 10^3^/μL, platelet enrichment ≥ 4-fold over whole blood, and leukocyte count ≤3 × 10^3^/μL, no detectable red blood cell (RBC) contamination. Only batches meeting these criteria were used for subsequent experiments. For the three independent PRP batches prepared in this study, the mean platelet concentration was 2.438 ± 0.244 × 10^6^/μL (4.72 ± 0.37-fold enrichment over whole blood), leukocyte count was 2.33 ± 0.58 × 10^3^/μL. The inter-batch coefficient of variation (CV) for platelet concentration was 7.84%, indicating high reproducibility of the preparation method.

The entire procedure was performed under sterile conditions. To obtain gel-like PRP for *in vivo* implantation, calcium gluconate solution (96 mg/mL) was added at a ratio of 10:1 (v/v), mixed thoroughly, and incubated at 37 °C for 30 min to allow PRP gel formation.

#### 2.1.3 Scanning electron microscopy (SEM) observation

The microstructure of the materials was examined using SEM. Samples were freeze-dried under vacuum for 48 h, then mounted on SEM stubs using conductive adhesive. The specimens were sputter-coated with gold at 10 mA for 120 s and subsequently observed under SEM. The micrographs were analyzed using ImageJ software.

## 3 In vitro experiments

### 3.1 Experimental animals

Three-week-old male Sprague-Dawley (SD) rats weighing approximately 40 g were used for the isolation of rBMSCs under protocols approved by the Ethics Committee of Nanjing University Kangda College.

### 3.2 Cell culture

Rat bone marrow mesenchymal stem cells (rBMSCs) were isolated from SD rats. Under sterile conditions, the bilateral hind limbs were disinfected, and the skin was incised with scissors. The femurs and tibias were dissected at the hip and ankle joints. Cruciform openings were created at both proximal and distal ends of the femurs and tibias. The bone marrow cavity was flushed with complete culture medium (L-DMEM: fetal bovine serum: penicillin/streptomycin = 89:10:1) using a sterile syringe inserted through the cruciform openings. The flushing process was repeated until the bone tissue turned white. The extracted rBMSCs were cultured in low-glucose DMEM (Gibco, United States of America) supplemented with 10% fetal bovine serum (FBS, Gibco) and 1% penicillin-streptomycin (Gibco) at 37 °C in a humidified atmosphere containing 5% CO_2_. Passage 3 (P3) rBMSCs were used for subsequent experiments.

### 3.3 Cell seeding and culture

Four experimental groups were established: (1) Control, in which rBMSCs were seeded directly onto tissue culture plastic; (2) MC, in which rBMSCs were seeded onto MC scaffolds; (3) PRP, in which PRP gel was prepared as described above prior to cell seeding; and (4) PRP + MC, in which MC scaffolds were immersed in PRP, incubated at 37 °C for 30 min, and then subjected to cell seeding.

Samples (10 mm in diameter, 2 mm in thickness) were sterilized by ^60^Co γ-irradiation at a dose of 25 kGy and placed in 48-well plates. rBMSCs were seeded onto the sample surfaces at a density of 2 × 10^4^ cells per well and cultured in L-DMEM (Gibco) containing 10% FBS and 1% penicillin-streptomycin at 37 °C with 5% CO_2_. Subsequent cell assays were performed at 1, 3, and 7 days.

### 3.4 Cell proliferation assay

At designated time points, cell-seeded samples were washed three times with PBS (pH 7.4) and fixed with 4% paraformaldehyde (Sigma-Aldrich) for 10 min. Cells were permeabilized with 0.1% (v/v) Triton X-100 (Sigma) for 10 min. F-actin was stained with tetramethylrhodamine isothiocyanate-conjugated phalloidin (TRITC-phalloidin, Sigma-Aldrich), and cell nuclei were stained with 4′,6-diamidino-2-phenylindole (DAPI, Sigma-Aldrich). Fluorescence imaging was performed using a confocal laser scanning microscope (Olympus FV10, Nagano, Japan).

At designated time points, cell-seeded samples were washed three times with PBS (pH 7.4) and digested with proteinase K (200 μL per well) overnight at 56 °C. DNA concentration was determined using the Quant-iT PicoGreen dsDNA Assay Kit (Invitrogen, Carlsbad, United States of America) according to the manufacturer’s instructions. A standard curve was prepared by serial dilution of a DNA stock solution (100 μg/mL) with 1× TE buffer to final concentrations of 0, 2, 20, 200, and 2000 ng/mL. The PicoGreen working solution was prepared by diluting the stock reagent 1:200 in 1× TE buffer. An equal volume (100 μL) of each cell lysate and PicoGreen working solution (100 μL) was mixed in a 96-well plate and incubated in the dark at room temperature for 5 min. A 200 μL aliquot of each reaction mixture was transferred to a microplate, and fluorescence intensity was measured using a BioTek Synergy four microplate reader at an excitation wavelength of 480 nm and an emission wavelength of 530 nm. DNA concentration in the cell lysate was calculated from the standard curve and expressed as ng/mL. Because all samples were processed using identical lysis volumes (200 μL per well) and equal aliquots (100 μL) were analyzed, DNA concentration (ng/mL) is directly proportional to the total DNA per well and was used as a relative measure of cell proliferation.

### 3.5 Osteogenic differentiation assessment

rBMSCs were seeded in 48-well plates at a density of 2 × 10^4^ cells per well. After 24 h of culture, the medium was replaced with osteogenic induction medium (containing 10 mM β-glycerophosphate, 100 nM dexamethasone, and 0.05 mM ascorbic acid). The medium was changed every 3 days. After 7 days of induction, samples were collected for alkaline phosphatase (ALP) staining and activity detection. After 21 days of induction, samples were collected for Alizarin Red S staining to assess extracellular matrix mineralization.

### 3.6 ALP staining and quantification

The culture medium was removed, and cells were washed twice with sterile PBS. Cells were fixed with 4% paraformaldehyde for 30 min, washed twice with PBS, and then incubated with 500 μL of ALP staining working solution per well in the dark at room temperature for 30 min. ALP staining was performed using a BCIP/NBT Alkaline Phosphatase Color Development Kit (Beyotime, China) according to the manufacturer’s instructions. The ALP staining working solution was prepared by mixing 5-bromo-4-chloro-3-indolyl phosphate (BCIP) and nitroblue tetrazolium (NBT) in the alkaline phosphatase buffer provided with the kit. The reaction was terminated by washing three times with ultrapure water. Images were captured using an optical microscope; the intensity of blue staining indicated ALP expression levels.

For quantitative analysis, the culture medium was removed, and cells were washed twice with PBS. Cell lysis buffer (RIPA lysis buffer, Beyotime, China, containing 1% PMSF) was added, and cells were lysed for 15 min. The lysates were collected into centrifuge tubes and centrifuged at 12,000 rpm for 5 min. The supernatants were retained, and ALP detection reagent (p-nitrophenyl phosphate substrate, Beyotime, China) was added. After thorough mixing, the samples were incubated at 37 °C for 10 min. The reaction was terminated with stop solution (0.5 M NaOH), and absorbance was measured at 405 nm using a microplate reader.

### 3.7 Alizarin Red S staining and quantification

Cells were gently washed twice with PBS to avoid dislodging calcium nodules. Cells were fixed with 1 mL of 4% paraformaldehyde per well at room temperature for 15 min, followed by two washes with deionized water. Alizarin Red S staining working solution was prepared by dissolving Alizarin Red S (Sigma-Aldrich, A5533) in distilled water at a concentration of 2% (w/v), adjusted to pH 4.1–4.3 with ammonium hydroxide. After two washes with deionized water to terminate the reaction, samples were observed under an optical microscope.

For quantitative analysis, excess liquid was removed from the wells. The Alizarin Red S stain was extracted from the mineralized nodules using 10% perchloric acid, and the plate was agitated on a shaker for 15 min until the calcium nodules on the material were completely dissolved. One hundred microliters of the solution were transferred to a 96-well plate, and absorbance was measured at 562 nm using a microplate reader to quantify bone calcium nodule formation.

### 3.8 Quantitative real-time PCR (qRT-PCR) analysis of osteogenic gene expression

rBMSCs were seeded onto the specimens at a density of 2 × 10^4^ cells per well. Following 24 h of adhesion, the culture medium was switched to osteogenic induction medium, consisting of low-glucose DMEM supplemented with 10% fetal bovine serum, 1% penicillin-streptomycin, 10 nM dexamethasone, 10 mM sodium β-glycerophosphate, and 0.05 mM ascorbic acid 2-phosphate. After 14 days of osteogenic differentiation, total RNA was extracted from the harvested cells using TRIzol® reagent (Invitrogen, United States of America) according to the manufacturer’s instructions. The concentration and purity of the extracted RNA were determined using a NanoDrop™ 2000 spectrophotometer (Thermo Scientific, United States of America), and 1 μg of total RNA was reverse-transcribed into complementary DNA (cDNA) using the PrimeScript™ RT Master Mix (Takara, Japan) in a 20 μL reaction volume.

qRT-PCR was performed on an ABI 9700 Real-Time PCR System (Applied Biosystems, United States of America) using Power SYBR® Green PCR Master Mix. The primer sequences were designed based on NCBI database searches and synthesized commercially. The primers for RUNX2 were: forward 5′-CAA​CCA​CAG​AAC​CAC​AAG​TGC-3′ and reverse 5′-AAA​TGA​CTC​GGT​TGG​TCT​CG-3′. Glyceraldehyde-3-phosphate dehydrogenase (GAPDH) served as the housekeeping gene (forward: 5′-TGG​GTG​TGA​ACC​ACG​AGA​A-3′; reverse: 5′-GGC​ATG​GAC​TGT​GGT​CAT​GA-3′). Each 20 μL PCR reaction contained 2 μL of 5-fold diluted cDNA and 0.4 μM of each primer. The thermal cycling protocol was initiated with a denaturation step at 95 °C for 5 min, followed by 40 cycles of 95 °C for 10 s and 60 °C for 1 min. A melting curve analysis was subsequently performed to verify the specificity of the amplification. Relative gene expression levels were quantified using the 2^−ΔΔCt method, with all samples normalized to the GAPDH housekeeping gene.

## 4 In vivo experiments

### 4.1 Animal experimental procedures

Male SD rats aged 8–12 weeks (weighing 250–300 g) were provided by the Laboratory Animal Research Center of Nanjing University Kangda College. Rats were randomly divided into four groups: blank control, PRP, mineralized collagen, and PRP + mineralized collagen.

### 4.2 Surgical procedure

Rats were weighed and anesthetized with 3% pentobarbital sodium (0.1 mL/100 g). After anesthesia, the surgical limb was shaved and disinfected with iodophor. A sterile drape was applied, and a lateral incision was made at the distal femur. Muscle tissue was dissected to expose the femoral condyle. A cylindrical bone defect (3 mm in diameter, 5 mm in depth) was created vertically on the lateral aspect of the femoral condyle using an electric drill. This defect dimension exceeds the critical size threshold for rat femoral condyle metaphyseal bone, which precludes spontaneous bone bridging during a 12-week observation period, as established in prior studies (Hou et al., 2023). After irrigation with normal saline, the bone defect was filled with cylindrical materials (3 mm in diameter, 5 mm in height) corresponding to the respective groups. The control group received normal saline treatment only. The muscle, fascia, and skin were sutured sequentially using 3–0 sutures. Rats were euthanatized at 4 and 12 weeks post-surgery. Femoral specimens were collected, fixed in 10% formaldehyde for 48 h, and subjected to Micro-CT scanning. Following scanning, all samples underwent decalcification, tissue embedding, and sectioning for histological evaluation of bone repair.

### 4.3 Micro-CT imaging analysis

Femur specimens were harvested from rats, fixed in 10% formaldehyde solution for 48 h, and then placed on the Micro-CT stage for scanning. The scanning parameters were set as follows: tube current 200 μA, tube voltage 85 kV, scanning range covering the entire specimen, resolution 10.150,115 μm, exposure time 384 m, and scanning angle 180°. After scanning, the raw image data were saved for subsequent analysis.

Three-dimensional reconstruction software NRecon (version 1.7.4.2, Bruker, Germany) was used to perform region of interest (ROI) reconstruction on the raw images. To optimize reconstruction quality and reduce image artifacts, the following parameters were adjusted: smoothing = 5, beam-hardening correction = 8, and ring artifact reduction = 25%. The ROI was strictly defined as a cylindrical volume corresponding to the original defect dimensions (3 mm diameter × 5 mm depth) centered on the defect site. After observing the X-, Y-, and Z-axis images, multi-planar and three-dimensional reconstructions were performed within the defined ROI. The reconstructed tomographic images were imported into Dataview software to observe and analyze the imaging manifestations of bone defect repair. Subsequently, the data were imported into CT Analyser software (version 2.7.0, Bruker, Germany) for quantitative analysis. A global thresholding method was applied to segment mineralized bone from non-mineralized tissue. The threshold value was determined by visual inspection of the grayscale histogram and applied uniformly across all specimens to ensure unbiased inter-group comparison. Using uniform parameters, the total volume (TV), bone volume (BV), bone volume fraction (BV/TV), trabecular number (Tb.N), bone mineral density (BMD), and trabecular separation (Tb.Sp) within the ROI were calculated. All image acquisition, reconstruction, and quantitative analysis were performed by an investigator blinded to group allocation. In addition, CTvox (version 2.7.0 r990, Bruker, Germany) was used for data reconstruction, and measurements were taken from the lateral femoral view for screenshot documentation.

### 4.4 Histological and immunohistochemical analysis

Femoral defect specimens were fixed in 10% formaldehyde for 48 h and decalcified with 10% ethylenediaminetetraacetic acid (EDTA, pH 7.4) at room temperature. The prepared samples were sectioned and stained with Masson’s trichrome and hematoxylin-eosin (H&E). Immunohistochemical staining for osteopontin (OPN) was performed on decalcified sections. After antigen retrieval in citrate buffer (pH 6.0) at 95 °C for 20 min, endogenous peroxidase activity was quenched with 3% H_2_O_2_ for 10 min. Sections were blocked with 5% goat serum and incubated overnight at 4 °C with primary antibody against OPN (Abcam, ab8448, 1:500). After washing, sections were incubated with horseradish peroxidase (HRP)-conjugated secondary antibody (goat anti-rabbit IgG, Abcam, ab205718, 1:1,000) for 1 h at room temperature. Immunoreactivity was visualized using a DAB chromogen kit (Vector Laboratories, United States of America). Sections were counterstained with hematoxylin, dehydrated, and mounted. Images were acquired using a microscope imaging system (Nikon E100 and DS-U3, Japan). Immunofluorescence images were captured using a fluorescence microscope (Nikon Eclipse C1 and Nikon DS-U3, Japan). Tissue growth and new bone formation in the femoral defect areas were analyzed using ImageJ software (version 1.53 S).

### 4.5 Statistical analysis

All data are expressed as mean ± standard deviation. For *in vitro* experiments, n = 3 represents independent biological replicates (separate cell cultures and scaffold batches), with each sample measured in technical triplicate. For the *in vivo* study, a total of 24 male SD rats were randomly allocated into four experimental groups (control, PRP, MC, and PRP + MC; n = 6 per group). Within each group, animals were further randomized to two sacrifice time points: 4 weeks (n = 3) and 12 weeks (n = 3). No animals were excluded from the analysis, and no mortality occurred during the observation period. Due to the small sample size (n = 3 per group), formal normality testing (e.g., Shapiro-Wilk) lacks sufficient power to reliably determine the distribution of the data. To adopt a conservative approach that avoids parametric assumptions, all comparisons among multiple groups were performed using the non-parametric Kruskal–Wallis test, followed by Dunn’s multiple comparisons test with Bonferroni correction. We acknowledge that the use of mean ± standard deviation alongside non-parametric testing is descriptive only, and all statistical inferences are based on rank-based comparisons. A two-tailed *P* value <0.05 was considered statistically significant. Statistical analyses were performed using GraphPad Prism software (version 10.0; GraphPad Software, San Diego, CA, United States of America).

## 5 Results

### 5.1 The preparation and microstructural characterization of PRP + MC composites

**FIGURE 1:**
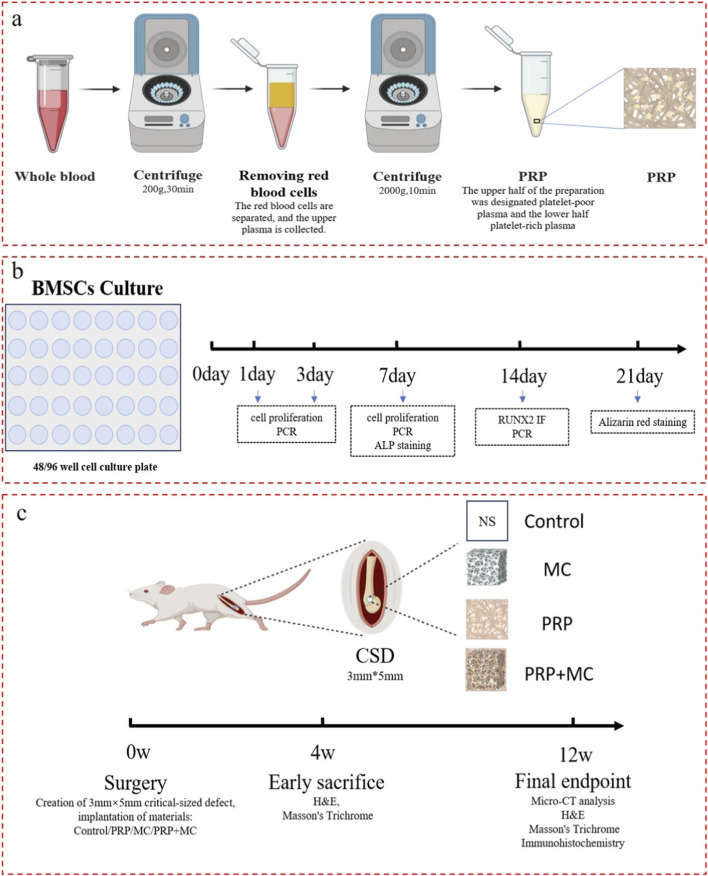
Schematic overview of PRP preparation and experimental design. **(a)** Platelet-rich plasma (PRP) was prepared from rat whole blood via a two-step centrifugation protocol (200 × g for 30 min, followed by 2000 × g for 10 min). **(b)** *In vitro* experimental workflow: rat bone marrow mesenchymal stem cells (rBMSCs) were seeded onto sterilized scaffolds in 48-well plates; cell proliferation was assessed at 1, 3, and 7 days, and osteogenic differentiation was evaluated at 7, 14, and 21 days. **(c)** *In vivo* experimental workflow: critical-sized cylindrical defects (3 mm in diameter, 5 mm in depth) were created in the lateral femoral condyle of male SD rats and randomly allocated to four treatment groups—normal saline (NS) control, PRP, mineralized collagen (MC), or PRP + MC composite. Bone regeneration was analyzed by Micro-CT and histological staining at 4 and 12 weeks post-implantation.

**FIGURE 2:**
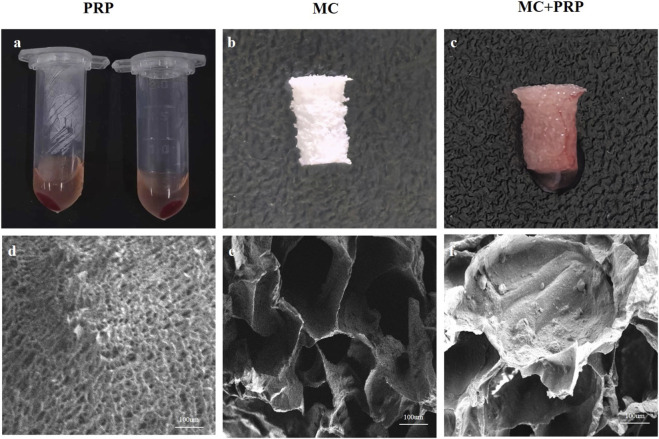
Preparation and microstructural characterization of PRP, MC, and PRP + MC composites. **(a)** PRP separated into distinct layers after two-step centrifugation of rat whole blood. **(b)** Macroscopic appearance of the mineralized collagen (MC) scaffold. **(c)** PRP + MC composite after gelation, exhibiting preserved cylindrical structure. **(d)** SEM image of PRP showing porous fibrin network morphology. **(e)** SEM image of MC displaying interconnected three-dimensional porous architecture. **(f)** SEM image of PRP + MC composite demonstrating fibrin coating on scaffold struts with maintained pore interconnectivity. Scale bars = 100 μm.

The preparation and microstructural characterization of PRP + MC composites are illustrated in Figure 1. Following a two-step centrifugation protocol, distinct stratification of rat whole blood was observed, with red blood cells sedimented at the bottom and the upper yellowish layer representing concentrated platelet-rich plasma (PRP) (Figure 2a). The mineralized collagen (MC) scaffold presented as a white porous cylinder with a foam-like texture (Figure 2b), while the PRP + MC composite, formed by immersing MC in PRP with subsequent calcium gluconate activation, exhibited a pinkish, semi-transparent appearance with preserved cylindrical macrostructural integrity (Figure 2c). SEM observation revealed that the MC scaffold possessed a highly interconnected porous architecture with uniform pore distribution (Figure 2d). The pore diameters ranged predominantly between 200 and 400 μm, with rough pore wall surfaces favorable for cell attachment. Higher magnification imaging further demonstrated the three-dimensional network structure with open, communicating pores essential for nutrient transport and vascular ingrowth (Figure 2e). In the PRP + MC composite group, SEM micrographs showed that the fibrin gel formed a thin, sheet-like coating over the MC struts and partially bridged the inter-pore spaces (Figure 2f). Despite the PRP coating, the underlying porous architecture remained largely preserved, with numerous open pores still visible. This indicates that the immersion strategy maintained the critical porous microenvironment of MC while introducing a bioactive PRP layer on the scaffold surface.

### 5.2 PRP + MC composite significantly enhances cytocompatibility and proliferation of rBMSCs

**FIGURE 3:**
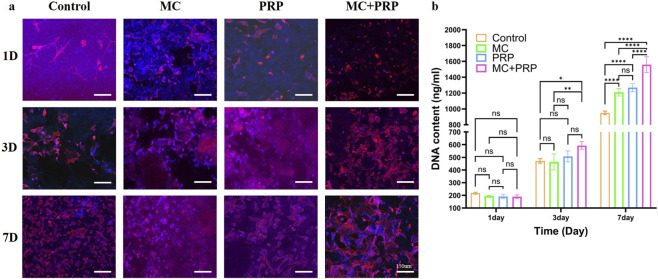
Cytocompatibility and proliferation of rBMSCs on different scaffolds. **(a)** Representative confocal laser scanning microscopy images of rBMSCs cultured on Control, MC, PRP, and PRP + MC scaffolds at 1, 3, and 7 days. F-actin was stained with TRITC-phalloidin (red) and nuclei with DAPI (blue). Scale bars = 150 μm. **(b)** Quantitative analysis of cell proliferation assessed by DNA content using the Quant-iT PicoGreen dsDNA Assay Kit at 1, 3, and 7 days. Data are expressed as mean ± standard deviation (n = 3 per time point). Statistical significance between groups was determined by the Kruskal–Wallis test followed by Dunn’s multiple comparisons test. *P < 0.05, **P < 0.01, ****P < 0.0001; ns, not significant.

To evaluate the cytocompatibility of the biomaterials, rBMSCs were seeded onto different substrates and observed at days 1, 3, and 7. Laser scanning confocal microscopy (LSCM) revealed that cells adhered and spread well on all material surfaces at each time point (Figure 3a). The cytoskeleton (F-actin, red fluorescence) exhibited typical spindle-shaped or polygonal morphologies, while nuclei (DAPI, blue fluorescence) appeared regular and uniformly distributed, with no evidence of cellular shrinkage or detachment, indicating excellent cytocompatibility and negligible cytotoxicity across all groups.

Cell proliferation kinetics demonstrated a time-dependent increase in DNA content across all groups (Figure 3b). At day 1, no significant differences were observed among groups (control, 216.7 ± 7.2 ng/mL; MC, 193.9 ± 6.6 ng/mL; PRP, 191 ± 15 ng/mL; PRP + MC, 190 ± 14 ng/mL; *P* = 0.063), suggesting comparable initial cell adhesion capacities. By day 3, the PRP + MC composite group exhibited the highest DNA content (592 ± 31 ng/mL), which was significantly greater than that of the control (472 ± 18 ng/mL; *P* < 0.01) and MC-alone (464 ± 64 ng/mL; *P* < 0.05) groups, whereas the difference versus the PRP-alone group (508 ± 43 ng/mL) did not reach statistical significance. Notably, at day 7, the PRP + MC group demonstrated significantly accelerated proliferation compared to all other groups (1,559 ± 100 ng/mL vs. control: 950 ± 22 ng/mL, MC: 1,211 ± 48 ng/mL, PRP: 1,269 ± 51 ng/mL; *P* < 0.01 for all comparisons). Additionally, both the MC-alone and PRP-alone groups exhibited significantly higher DNA content than the control group (*P* < 0.01), whereas no significant difference was detected between the MC and PRP groups (*P* > 0.05). These findings indicate that the PRP + MC composite system exerts an enhanced proliferative effect compared with either component alone during the later culture period (day 7), likely reflecting both pro-adhesive ECM properties and mitogenic stimulation, whereas single materials provided only basal support during early cultivation.

### 5.3 PRP + MC composite promotes osteogenic differentiation of rBMSCs *in vitro*

To assess the osteoinductive capacity of the materials, early osteogenic differentiation was evaluated by ALP staining and activity assay at day 7, while extracellular matrix mineralization was assessed by Alizarin Red S staining and quantification at day 21.

**FIGURE 4:**
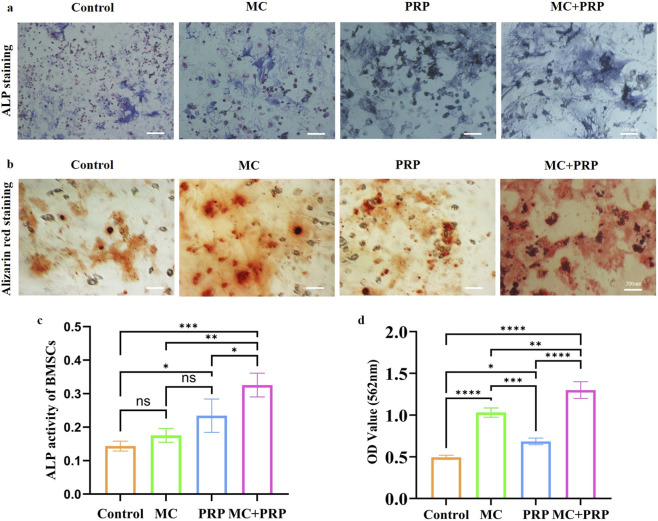
Osteogenic differentiation of rBMSCs on different scaffolds. **(a)** Representative bright-field images of alkaline phosphatase (ALP) staining at day 7 following osteogenic induction. Scale bars = 200 μm. **(b)** Representative bright-field images of Alizarin Red S staining at day 21 showing extracellular matrix mineralization. Scale bars = 200 μm. **(c)** Quantitative analysis of ALP activity. **(d)** Quantitative analysis of mineralized nodule formation assessed by Alizarin Red S extraction and absorbance measurement at 562 nm. Data are expressed as mean ± standard deviation (n = 3 per time point). Statistical significance between groups was determined by Kruskal–Wallis test followed by Dunn’s multiple comparisons test. *P < 0.05, **P < 0.01, ***P < 0.001, ****P < 0.0001; ns, not significant.

Early osteogenic marker expression: As shown in Figure 4a, ALP staining revealed varying degrees of blue-purple positive staining across all groups, confirming osteophenotypic differentiation. The PRP + MC composite group exhibited the most intense and extensive blue-purple coloration, with significantly greater coverage area compared to other groups. The MC-alone group showed weaker and heterogeneous staining, while the control group displayed only scattered, faint positive signals. Quantitative analysis (Figure 4c) demonstrated that ALP activity in the PRP + MC group was significantly higher than that in the control (0.326 ± 0.036 vs. 0.143 ± 0.015; *P* < 0.001), MC-alone (0.326 ± 0.036 vs. 0.175 ± 0.021; *P* < 0.01), and PRP-alone groups (0.326 ± 0.036 vs. 0.234 ± 0.050; *P* < 0.05). Additionally, the PRP-alone group exhibited significantly elevated ALP activity compared to the control (0.234 ± 0.050 vs. 0.143 ± 0.015; *P* < 0.05), whereas no significant difference was observed between the MC-alone and control groups (0.175 ± 0.021 vs. 0.143 ± 0.015; *P* > 0.05) or between the MC-alone and PRP-alone groups (0.175 ± 0.021 vs. 0.234 ± 0.050; *P* > 0.05).

Extracellular matrix mineralization: Alizarin Red S staining at day 21 (Figure 4b) revealed dense, deep-red mineralized nodules in the PRP + MC composite group, characterized by numerous, large-volume deposits with partial confluent sheet-like distribution. The MC-alone group exhibited scattered red nodules with reduced quantity and intensity compared to the composite group. The PRP-alone group showed only small, faintly stained punctate deposits, while the control group lacked discernible mineralized nodules. Quantitative analysis (Figure 4d) confirmed that the PRP + MC composite group exhibited significantly higher absorbance values (562 nm) compared to the control (1.30 ± 0.10 vs. 0.493 ± 0.025; *P* < 0.0001), MC-alone (1.30 ± 0.10 vs. 1.030 ± 0.056; *P* < 0.01), and PRP-alone groups (1.300 ± 0.10 vs. 0.683 ± 0.040; *P* < 0.0001), with mineralization approximately 1.9-fold higher than that of the PRP-alone group. Significant differences were also detected between the MC-alone and PRP-alone groups (1.030 ± 0.056 vs. 0.683 ± 0.040; *P* < 0.001), as well as between the MC-alone and control groups (*P* < 0.0001) and the PRP-alone and control groups (0.683 ± 0.040 vs. 0.493 ± 0.025; *P* < 0.05).

These results demonstrate that the PRP + MC composite system markedly enhances both early osteogenic differentiation (upregulated ALP expression) and late-stage mineralized matrix formation. While MC serves as an osteoconductive scaffold facilitating calcium deposition, the addition of PRP substantially amplifies this effect.

### 5.4 PRP + MC composite upregulates osteogenic-specific marker expression in rBMSCs

To further validate the pro-osteogenic effect, quantitative real-time PCR (qRT-PCR) were performed at day 14 to examine the mRNA levels of RUNX2, the master transcription factor regulating osteogenesis.

**FIGURE 5:**
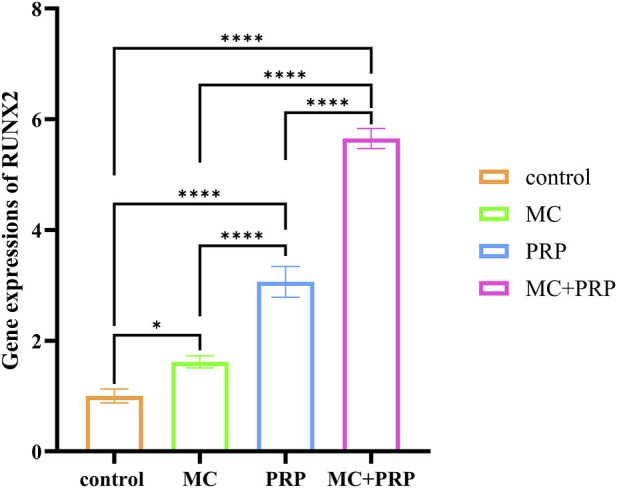
Expression of RUNX2 in rBMSCs cultured on different scaffolds at day 14. Quantitative real-time PCR (qRT-PCR) analysis of RUNX2 mRNA expression normalized to the housekeeping gene and presented as fold change relative to the control group. Data are expressed as mean ± standard deviation (n = 3 per time point). Statistical significance between groups was determined by the Kruskal–Wallis test followed by Dunn’s multiple comparisons test. *P < 0.05, ****P < 0.0001.

Gene transcription analysis (Figure 5) demonstrated that RUNX2 mRNA expression in the PRP + MC group was significantly higher than in the MC-alone (5.65 ± 0.18-fold vs. 3.06 ± 0.28-fold; *P* < 0.0001), PRP-alone (5.65 ± 0.18-fold vs. 1.62 ± 0.11-fold; *P* < 0.0001), and control groups (5.65 ± 0.18-fold vs. 1.00 ± 0.13-fold; *P* < 0.0001), approximately 5.6-fold greater than the control. Both the MC-alone (3.06 ± 0.28-fold vs. 1.00 ± 0.13-fold; *P* < 0.0001) and PRP-alone (1.62 ± 0.11-fold vs. 1.00 ± 0.13-fold; *P* < 0.01) groups exhibited significantly elevated RUNX2 expression compared to the control, though lower than the composite group. These results confirm that the PRP + MC composite system significantly activates osteogenic differentiation at the transcriptional level. Collectively, these findings indicate that PRP + MC biomaterials effectively promote osteophenotypic differentiation of rBMSCs, likely through activation of RUNX2-mediated osteogenic signaling pathways.

### 5.5 PRP + MC composite promotes repair of critical-sized femoral defects in rats

To evaluate the bone regeneration capacity *in vivo*, critical-sized cylindrical femoral condyle defects (3 mm diameter, 5 mm depth) were created and analyzed by Micro-CT at 12 weeks post-implantation.

**FIGURE 6:**
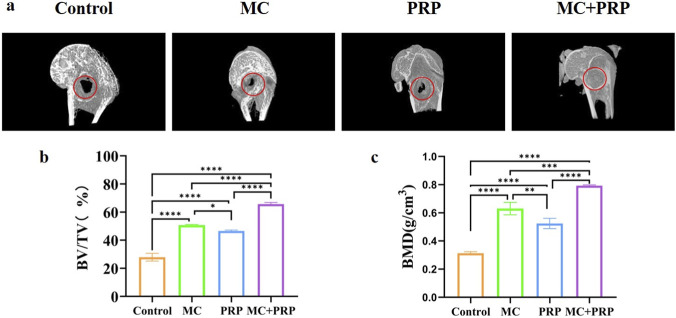
Micro-CT analysis of bone regeneration in critical-sized femoral condyle defects at 12 weeks post-implantation. **(a)** Representative three-dimensional Micro-CT reconstructions of the distal femur showing defect areas (red circles) in the Control, MC, PRP, and PRP + MC groups. **(b,c)** Quantitative morphometric analysis of bone volume fraction (BV/TV) **(b)**, bone mineral density (BMD) **(c)**. Data are expressed as mean ± standard deviation (n = 3 per time point). Statistical significance between groups was determined by the Kruskal–Wallis test followed by Dunn’s multiple comparisons test. *P < 0.05, **P < 0.01, ***P < 0.001, ****P < 0.0001.

Three-dimensional imaging: As shown in Figure 6a, the non-healing nature of this defect model was confirmed by the control group, which exhibited persistent defect gaps with minimal bone ingrowth (BV/TV = 27.9 ± 2.7%) at 12 weeks, consistent with established critical-sized defect criteria (Hou et al., 2023). The PRP-alone group demonstrated partial callus formation with low-density centers remaining unhealed. The MC-alone group showed near-complete defect filling, though with heterogeneous mineral density distribution. In contrast, the PRP + MC composite group achieved extensive defect filling with high-density bone tissue, well-organized trabecular architecture, and seamless integration with host bone, indicating advanced structural restoration.

Quantitative morphometric analysis (Figures 6b,c): The PRP + MC composite group achieved a bone volume fraction (BV/TV) of 65.7% ± 1.2%, significantly superior to the MC-alone group (50.73% ± 0.39%), the PRP-alone group (46.54% ± 0.68%), and the control group (27.9% ± 2.7%) (*P* < 0.0001). Bone mineral density (BMD) analysis revealed significantly superior mineralization in the PRP + MC group (0.793 ± 0.005 mg HA/cm^3^) compared to all other groups (MC: 0.630 ± 0.044; PRP: 0.525 ± 0.037; control: 0.313 ± 0.011; *P* < 0.01 vs. MC, *P* < 0.001 vs. PRP, *P* < 0.0001 vs. control), with values approaching those of surrounding host bone, suggesting functional tissue restoration.

These results demonstrate that PRP + MC biomaterials exhibit superior osteoinductive and osteoconductive effects, significantly accelerating both structural and functional healing of critical-sized bone defects.

### 5.6 PRP + MC composite promotes histological healing and maturation of bone defects

To comprehensively assess the histological characteristics of bone regeneration, decalcified sections were prepared at 4 and 12 weeks post-surgery for hematoxylin-eosin (H&E), Masson’s trichrome staining, and immunohistochemical analysis. Semi-quantitative image analysis was employed to evaluate new bone formation area and target protein expression.

**FIGURE 7:**
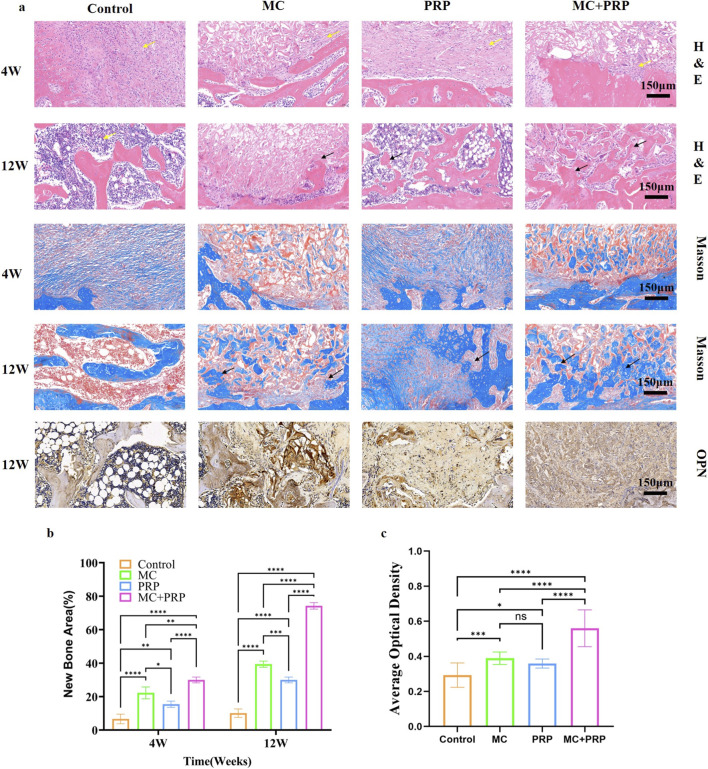
Histological evaluation of bone defect healing at 4 and 12 weeks post-implantation. **(a)** Hematoxylin-eosin (H&E) staining at 4 weeks and 12 weeks; black arrows indicate new bone formation; yellow arrows show inflammatory infiltrates. Masson’s trichrome staining at 4 weeks and 12 weeks; black arrows indicate new bone formation. Immunohistochemical staining for osteopontin (OPN) at 12 weeks. Scale bars = 150 μm. **(b)** Quantitative analysis of new bone area percentage at 4 and 12 weeks. **(c)** Quantitative analysis of average optical density (AOD) for OPN expression at 12 weeks. Data are expressed as mean ± standard deviation (n = 3 per group per time point). Statistical significance between groups was determined by the Kruskal–Wallis test followed by Dunn’s multiple comparisons test. *P < 0.05, **P < 0.01, ***P < 0.001, ****P < 0.0001; ns, not significant.

Histomorphological observations (Figure 7a): At week 4, the control group defects were predominantly filled with fibrous connective tissue and inflammatory infiltrates (indicated by yellow arrows), with nascent bone limited to defect margins as sparse woven bone. The MC-alone group showed residual material surrounded by limited new bone formation (indicated by black arrows), characterized by thin, irregularly arranged trabeculae. The PRP-alone group exhibited reduced fibrous components but limited bone matrix deposition. In contrast, the PRP + MC composite group displayed extensive new bone formation with interconnected immature bone structures; marrow cavity-like structures began forming with synchronized material degradation and bone ingrowth.

By week 12, the control group remained dominated by fibrous scar tissue without mature lamellar bone in the defect center. The MC group showed increased bone area but persistent undegraded material particles and fibrous septa. The PRP group exhibited increased matrix density but disorganized trabecular architecture. Conversely, the PRP + MC composite group demonstrated complete defect filling with trabecular-like bone arranged in irregular patterns, early Haversian canal-like structures, and apparent host bone integration at the defect margins. Masson’s trichrome staining revealed dense, directionally organized blue collagen fibers, indicating high tissue maturation.

Quantitative analysis of new bone formation (Figure 7b): Image analysis revealed time-dependent increases in new bone area percentage across all groups. At week 4, the PRP + MC composite group exhibited significantly higher new bone area compared to the control (30.0% ± 1.7% vs. 6.7% ± 2.9%; *P* < 0.0001), PRP-alone (30.0% ± 1.7% vs. 15.5% ± 1.9%; *P* < 0.001), and MC-alone groups (30.0% ± 1.7% vs. 22.3% ± 3.5%; *P* < 0.05), approximately 4.5-fold greater than the control. Both the MC-alone (22.3% ± 3.5% vs. 6.7% ± 2.9%; *P* < 0.001) and PRP-alone (15.5% ± 1.9% vs. 6.7% ± 2.9%; *P* < 0.05) groups were significantly superior to the control, whereas no significant difference was detected between the MC and PRP groups (*P* = 0.051). By week 12, the PRP + MC group achieved the highest new bone area (74.2% ± 2.0%), significantly exceeding the MC group (39.4% ± 1.8%) and PRP group (30.0% ± 1.7%) (*P* < 0.0001 for all comparisons), approaching normal trabecular bone levels, while the control group remained minimal (10.1% ± 2.5%).

Osteogenesis-related protein expression (Figure 7c): Immunohistochemical analysis of osteopontin (OPN) revealed that the PRP + MC composite group exhibited significantly higher average optical density (AOD) compared to the control (0.56 ± 0.11 vs. 0.293 ± 0.070; *P* < 0.0001), MC-alone (0.56 ± 0.11 vs. 0.389 ± 0.036; *P* < 0.0001), and PRP-alone groups (0.56 ± 0.11 vs. 0.360 ± 0.026; *P* < 0.0001). Specifically, the MC-alone group was significantly higher than the control (*P* < 0.001), and the PRP-alone group was also significantly higher than the control (*P* < 0.05), whereas no significant difference was detected between the PRP-alone and MC-alone groups (*P* > 0.05). The PRP + MC composite demonstrated significant upregulation compared to both single-component groups, indicating combined enhancement at the protein expression level.

These histological findings confirm that PRP + MC biomaterials facilitate bone defect repair through enhanced osteogenic differentiation.

## 6 Discussion

Given the limitations of autologous bone grafting—the established “gold standard”—including restricted donor availability and inability to meet complex clinical demands, alongside the risks of disease transmission and immune rejection associated with allogeneic bone, the development of optimal bone substitute materials remains a critical challenge in orthopedics and maxillofacial surgery (Liu et al., 2025; Tian et al., 2024). This study demonstrates, through both *in vitro* and *in vivo* experiments, that the combination of platelet-rich plasma (PRP) and mineralized collagen (MC) creates an additive or enhanced effect that promotes osteogenic differentiation of bone marrow mesenchymal stem cells (BMSCs) and enhances bone regeneration in critical-sized defect models.

MC, as a biomimetic composite of type I collagen and nano-hydroxyapatite (nHA), features a periodically ordered nanostructure that replicates the chemical composition and microarchitecture of native bone extracellular matrix, providing essential mechanical support for cell adhesion and migration (Zhu W. et al., 2023). However, MC lacks osteogenic cells and exhibits relatively slow osteogenesis, leading to clinical concerns regarding delayed material degradation and incomplete defect healing (Gao et al., 2020)—challenges common to artificial bone substitutes (Kolliopoulos and Harley, 2024). This limitation is evidenced in our study by the ALP staining results at day 7, wherein the MC-alone group showed no significant advantage over the PRP-alone group and demonstrated no statistically significant difference compared to the blank control. Therefore, incorporating growth factors into artificial bone scaffolds represents a crucial strategy to enhance therapeutic efficacy (Rambhia et al., 2024).

PRP contains a cocktail of growth factors, including PDGF, TGF-β1, VEGF, and IGF-1, which have been applied in bone defect treatment (Honda et al., 2013; Gianakos et al., 2015). These factors collectively activate cell cycle-related signaling pathways, promote mitosis, and rapidly induce cell adhesion, migration, and osteoblastic differentiation (Abdel-Haffiez and Khalil, 2024). Unlike recombinant human BMP, which has failed to demonstrate sustained significant advantages in randomized controlled clinical trials and carries risks of ectopic bone formation, excessive inflammatory responses, and high costs associated with supraphysiological dosing, PRP offers a multi-factorial approach with an excellent safety profile (Hasenbein et al., 2022). Furthermore, the fibrin matrix within PRP not only provides immediate hemostasis but also serves as a protective reservoir for growth factors, shielding them from proteolytic degradation and extending their bioavailability within the MC scaffold, while PRP preparation remains straightforward and cost-effective compared to recombinant protein manufacturing (Wu et al., 2025). Our ALP staining results confirmed that PRP promotes osteogenic differentiation of BMSCs more effectively than MC alone or the control. However, PRP alone faces challenges regarding rapid diffusion and poor structural integrity, particularly in large bone defects where effective defect filling is difficult (Nair et al., 2009). This limitation was further validated in our study through long-term Alizarin Red staining and subsequent histological and CT analyses, wherein the PRP-alone group demonstrated inferior bone repair capacity compared to the MC-alone group.

Current strategies for loading bioactive factors into MC include: (1) immersion in aqueous factor (AF) solutions for efficient adsorption; (2) heparin-modified MC surfaces with affinity for AF; (3) incorporation of polymers via injection or mixing; and (4) functionalized mineralized collagen (FMC) produced by mixing AF with MC precursors (Zhu et al., 2023b). In this study, we employed an immersion strategy, submerging MC in PRP, primarily because this approach aligns with clinical workflows where PRP is typically prepared from patient blood via centrifugation (Anaya-Sampayo et al., 2024). This method preserves the porous architecture of MC, maintaining pore diameters within the critical range of 200–400 μm—essential for providing an optimal microenvironment for cell attachment, proliferation, and differentiation while permitting capillary ingrowth to meet metabolic demands (Wang et al., 2022). With PRP coating the MC surface, multiple growth factors can be effectively released (Han et al., 2023). Confocal microscopy confirmed that rBMSCs adhered and spread well on all material surfaces with normal morphology and no apparent cytotoxicity. DNA quantification further demonstrated that the PRP + MC composite group exhibited significantly enhanced cell proliferation at day 7 compared to single-material groups and controls, suggesting a combined beneficial effect between PRP and MC in promoting cellular proliferation.

As demonstrated by do Amaral et al., collagen-glycosaminoglycan scaffolds functionalized with activated PRP—forming a fibrin gel homogeneously within the pores—sustained the release of key growth factors (FGF, TGFβ, VEGF, and PDGF) for up to 14 days *in vitro*, enhanced mechanical properties, and supported proliferation of human endothelial cells, mesenchymal stromal cells, fibroblasts, and keratinocytes even without serum supplementation (do Amaral et al., 2019). We hypothesize that a similar prolonged release profile contributes to the sustained activation of osteogenic master genes such as RUNX2, rather than transient induction (Katsianou et al., 2025). Such sustained release avoids supraphysiological concentration peaks associated with single-dose exogenous growth factor administration, maintaining a persistent concentration gradient of osteogenic factors within the defect microenvironment (Jiang et al., 2021). As Zhang et al. noted, PDGF-BB and FGF-B in PRP not only directly promote BMSCs proliferation but also enhance cell survival and differentiation through activation of the PI3K/AKT/mTOR signaling pathway (Zhang et al., 2025; Liu et al., 2019).

Growth factors in PRP are susceptible to degradation by proteases (e.g., MMPs, plasmin). The weakly crystalline nano-HA within MC not only provides mechanical support but may competitively inhibit protease activity through Ca^2+^-dependent binding to active sites, thereby protecting growth factors from degradation (Censi et al., 2020; Chen et al., 2025; Huang et al., 2023). Surface Ca^2+^ ions can bind to carboxyl/amino groups of growth factors, creating localized high-concentration signaling gradients that prolong bioactivity (Özcan and Çiftçioğlu, 2021; Chen et al., 2021). More importantly, this biomimetic mineralized microenvironment may regulate the epigenetic status of BMSCs (Chen et al., 2021). In our study, the PRP + MC group exhibited a 5.6-fold upregulation of RUNX2 mRNA expression. This sustained signal activation of the master transcriptional regulator for osteogenic differentiation subsequently initiates downstream expression cascades of Osterix and osteocalcin—mechanisms further corroborated by our immunohistochemical analysis demonstrating high OPN expression. Fibrinogen adsorbed onto nHA surfaces forms a protective coating that maintains growth factor conformational integrity (Li et al., 2021; Wu et al., 2023). This “biomolecular corona” effect ensures sustained release of active factors from PRP over 2–4 weeks post-implantation; VEGF and PDGF within PRP promote vascular endothelial cell migration, improving oxygen and nutrient supply within the MC interior and maintaining homeostasis of the mineralized microenvironment (Kataoka et al., 2021; Al-Hamed et al., 2021; Gruber, 2000). While we hypothesize that enhanced vascularization may have contributed to the observed bone regeneration, this study did not directly evaluate neovascularization. Therefore, any inference regarding H-type vessel-mediated ‘angiogenesis-osteogenesis’ coupling remains speculative. Future investigations must include direct vascular assessment to validate the role of angiogenesis in PRP + MC mediated bone repair.

This study has several limitations. First, the present study employed a relatively small sample size (n = 3), which is a limitation that may constrain the generalizability of our findings. However, this sample size is consistent with established preclinical protocols for rat femoral condyle critical-sized defect models, where group sizes of three to five animals are frequently employed for initial proof-of-concept studies using Micro-CT and histomorphometric endpoints (Hou et al., 2023). Long-term studies (24–52 weeks) combined with biomechanical testing (three-point bending or nanoindentation) are essential to confirm whether regenerated tissue achieves functional mechanical strength comparable to native bone. Second, the specific contributions of exosomes or extracellular vesicles within PRP to the observed osteogenic effects remain undefined. Recent evidence suggests that platelet-derived exosomes may mediate paracrine signaling through miRNA transfer; isolating and testing PRP exosomes separately could identify key bioactive cargo and potentially enable cell-free therapeutic strategies. Third, while RUNX2 has been identified as a core mediator of osteogenic differentiation in previous studies (Katsianou et al., 2025), upstream signaling pathways (e.g., Wnt/β-catenin, MAPK/ERK) and their interactions with the scaffold microenvironment were not extensively investigated. Single-cell RNA sequencing of cells harvested from defect sites at multiple timepoints would provide an unbiased, high-resolution atlas of cellular heterogeneity and intercellular communication networks, offering deeper insights into the mechanistic underpinnings of PRP + MC mediated bone regeneration. Fourth, the present study did not include biomechanical testing to assess the functional mechanical properties of the regenerated bone at 12 weeks. While Micro-CT and histomorphometry provide enhanced structural and morphological evidence of bone formation, they do not directly evaluate whether the regenerated tissue has achieved mechanical strength comparable to native bone. This is a significant limitation for claims of “robust regeneration” and functional restoration. Future studies should incorporate biomechanical analysis at 24–52 weeks to confirm that the PRP + MC composite achieves load-bearing capacity sufficient for clinical translation.

## 7 Conclusion

In conclusion, this study establishes that PRP + MC represents a biomimetic and cost-effective strategy for enhancing bone repair. Through the integration of structural scaffolding and biological stimulation, this composite system activates RUNX2-mediated osteogenic signaling pathways, achieving enhanced bone regeneration in critical-sized defects. These findings position PRP + MC as a promising preclinical strategy that warrants further validation through larger animal studies, biomechanical testing, and long-term remodeling assessment before clinical application. Future investigations should focus on elucidating immunomodulatory mechanisms, characterizing long-term remodeling dynamics, and establishing standardized preparation protocols to facilitate clinical translation.

## Data Availability

The raw data supporting the conclusions of this article will be made available by the authors, without undue reservation.
